# Optimization of buffer solutions to analyze inflammatory cytokines in gingival crevicular fluid by multiplex flow cytometry

**DOI:** 10.4317/medoral.19852

**Published:** 2014-06-01

**Authors:** María-Judith Ríos-Lugo, Conchita Martin, José-Antonio Alarcón, Ana Esquifino, Germán Barbieri, Patricia Solano, Mariano Sanz

**Affiliations:** 1 Department of Stomatology III, Area of Periodontics, Complutense University of Madrid, Madrid, Spain; 2 Department of Stomatology IV, Area of Orthodontics, Complutense University of Madrid, Madrid, Spain; 3 Department of Stomatology, Area of Orthodontics, School of Dentistry. University of Granada, Granada, Spain; 4 Department of Biochemistry and Molecular biology III, Medicine School, Complutense University of Madrid, Madrid, Spain

## Abstract

Objective: the aim of this study was to test two buffer solutions in order to attain a reliable and reproducible analysis of inflammatory cytokines (IL-1β, IL-6, TNF-α, OPG, OPN and OC), in gingival crevicular fluid (GCF) by flow cytometry. Material and Methods: GCF samples from healthy volunteers were collected with perio-paper strips and diluted either in phosphate buffered saline (PBS) or Tris-HCl buffer, with and without protease inhibitors (PI). Cytokine immunoassays were carried out by flow cytometry (Luminex Xmap 200) generating standard curves. Results: standards curves generated with the use of phosphate-buffered saline (PBS) demonstrated best adjustment for cytokines IL-1ß, IL-6 and TNF- α levels, when using Tris-HCl (*p*<0.05). Conclusions: The use of PBS buffer with the addition of PI provided reliable measurements of inflammatory biomarkers in GCF samples of healthy volunteers.

** Key words:**Curve fitting, flow cytometer, immunoassay buffer, crevicular fluid, cytokines.

## Introduction

The analysis of immunological biomarkers in GCF has been mostly used for research purposes, although it has also been considered appropriate for a periodontal disease diagnosis and the evaluation of the patient’s response to therapy (1). Among biomarkers, cytokines involved in the cellular inflammatory response (2) have been recognized as potentially useful diagnostic or prognostic biomarkers of periodontal destruction (3). In the quantification of these cytokines in GCF, different analytical methods have been used providing very heterogeneous results (4). Different factors have been attributed to justify this heterogeneity including: the sampling method, the contamination of the sample with oral fluids, the buffer solution used to sample dilution and maintenance condition, degradation of GCF proteases, and the different cytokine identification methods (4,5). Although most studies have used the filter paper strips to collect GCF, this technique requires the selection of the appropriate sites, the careful placement of the periopaper strips, the avoidance of any fluid contamination and the suitable calibration of the fluid volume measuring device (Periotron®) (6,7). Moreover, since the amount of fluid collected is usually very small, mostly in sites without a clear inflammatory status (1-2 µL), the assay sensitivity used for the cytokine detection may be compromised (8). It is, therefore, important, to find out the proper buffer to dilute the samples (usually in volumes of 100-150 ml) that does not interfere with the cytokine detection. Buffer solutions, due to their chemical properties, may interact with the biochemical components present in samples, or they may interfere with non-protein substances, thus altering the results, mainly with the use of immunoassay techniques (9). Some important considerations when choosing a buffer solution are the pH, the salts and detergents. The pH of buffers; can affect protease activity solubility can be increased with a moderate amount of salts; and the use of any charged detergents will interfere with the analysis. PBS buffer may help to minimize these variations and give protein uniformity, maintaining a constant pH and presenting the osmolality levels and ion concentrations of the solution which usually match those of the human body (isotonic) (10). On the other hand, Tris-HCl, because of its nature, possibly causes important variability and interactions between proteins and different amine groups resulting in high background and false signals. Proteins from GCF samples need to be extracted efficiently and without degradation to make the best use of a limited resource. However, protein extraction inevitably compromises preservation. In order to avoid proteolytic degradation, protease inhibitors are added to ensure protein preservation (5), although they regulate different enzymatic reactions, such as proteolysis of proteins, proteolysis of phosphatases converted into proteolytic substrates, which may have catalytic responses and probably result in high degradation of samples. In research, enzyme-linked immunosorbent assay (ELISA) and the ELISpot® have been the most commonly used analytical methods, based on the analysis of each cytokine individually. This demands arduous work and requires enough fluid volume in the sample to provide aliquots for each analyte (11). Recently, the introduction of bioassays allowing the simultaneous assessment of multiple analytes, such as by multiplexing, have solved many of these drawbacks, although research findings require the appropriate validation and standardization, especially when used with clinical samples (12). The FDA guidelines state the importance of the adequate validation of the tests assessing biomarkers used in a patient’s diagnosis in order to ensure their possible clinical benefit (13,14). The Luminex Xmap200 (flow cytometer technique) is a new diagnostic method, which has been used in laboratory assessment of cytokines and hormones (15), allowing up to 20 cytokine targets to be measured from one single sample.

The aim of this study is to validate the reliability of a 7-analyte multiplex assay (Luminex Xmap200) by comparing two different common buffers used for diluting GCF samples, with or without the addition of protease inhibitors.

## Material and Methods

- Subjects

11 healthy volunteers were selected from the Faculty of Dentistry of the Complutense University of Madrid, Spain. Subjects were informed about the objectives of this study and they agreed to take part in it by providing written informed consent prior to the sampling collection process.

- Sampling

Three GCF samples were collected from each subject from the disto-buccal site of tooth 11 and the mesio-buccal site of teeth 21 and 22. These samples were randomly assigned to the three following dilution buffer assignments: Tris, Tris+PI and PBS+PI. Another 5 samples of a healthy volunteer were collected from disto-buccal (teeth 12 and 42) and mesio-buccal (teeth 21, 31 and 32) sites to make up the fourth group, the PBS group. Upper and lower anterior teeth were selected in order to avoid sample contamination with oral fluids such as saliva or blood.

Perio® paper strips (Oral Flow Inc., #593520) were used to collect GCF samples using the following technique : once the sites were isolated with cotton rolls and gently air-dried, the Perio® paper strips were inserted in the gingival sulcus for 30 seconds. The paper strips were then inserted in the Periotron 8000® device (Harco, Tustin, CA, USA), previously calibrated to each individual sample to obtain the fluid volume. Following the randomization pattern described before, the strips were placed in the vials containing the studied buffer solutions (Tris, Tris+PI, PBS+PI and PBS) and were centrifuged at 12,500 rpm for 5 minutes at 4°C. The supernatants obtained were stored frozen at -80°C until assayed.

- Immunoassay procedure 

Before starting the bioassay, the samples were thawed on ice and once ready for use, they were spun for 10 seconds and centrifuged at 1500 rpm for 15 seconds. Cytokine profiles were obtained using the X map200 device and processed through the commercial human Luminex® kits (Millipore, Watford, UK) following the manufacturer’s protocol . In summary, the wells of a 1.2 μm filter bottom 96-well microtiter plates were pre-wetted with assay buffer, then 25μL of the sample and 25 µL of the selected buffer (PBS or Tris-HCl with or without PI) were added to appropriate background, standards and control wells according to the bioassay standard curve. Plates were then incubated in 25 μL of premixed microbeads overnight on an orbital shaker at 4°C, washed twice with wash buffer and then, 25 μL of biotinylated detector antibody was added to each well, and incubated for 1 h at room temperature. Without further washing, 25 µL of streptavidinp-hycoerythrin solution was added to each well, and the plates were incubated for 30 minutes at room temperature on a plate shaker, protected from direct light. Before the analysis, microbeads were washed twice in wash buffer and suspended in 100 μL/well of Luminex sheath fluid.

- Standard curve preparation 

To prepare the antibody-immobilized beads, the bead diluent (provided in the Millipore kit) was pre-warmed to room temperature, sonicated and shaken for 1 minute. This bead diluent together with 150 μL of analyte beads were added to the Bead Mixing Vial until obtaining 3.0 mL of diluted beads. Serial dilutions of standards in deionized water were performed (0- Background). The levels (pg/mL) of the obtained standard curve are shown in  Table 1. Before use, quality controls for each cytokine were reconstituted with 250 µL of deionized water, transferred to polypropylene tubes and allowed to settle for 5-10 min.

**Table 1:**
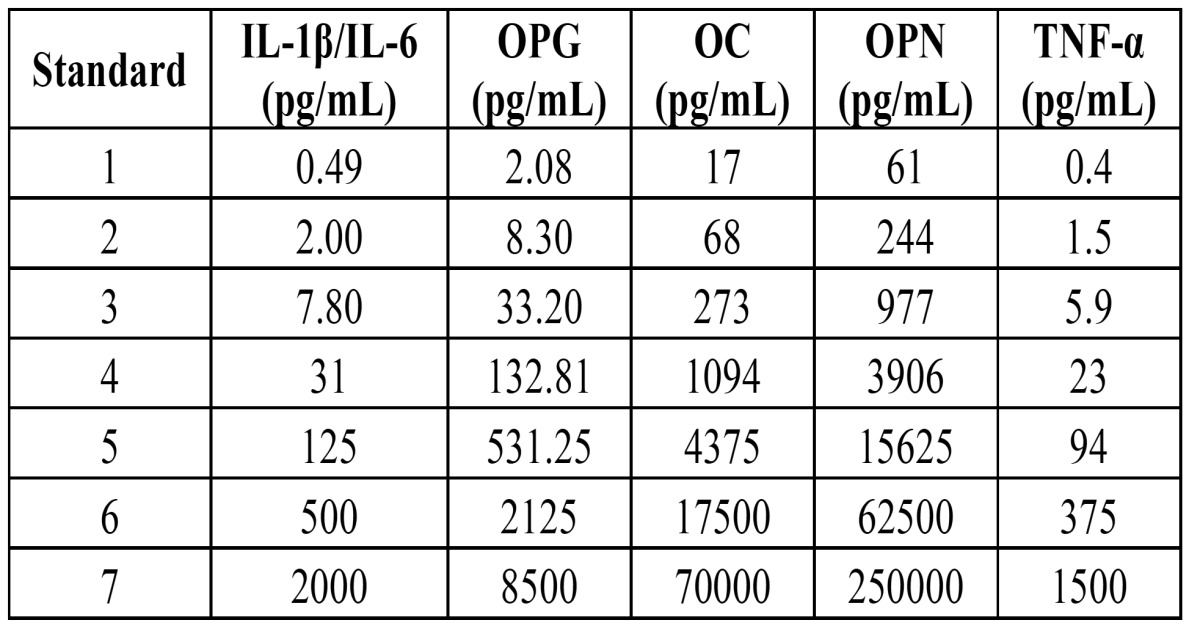
Standard curves of biomarkers for Luminex Xmap 200.

- Statistical Analysis

The analysis included assessment of the buffer effect (i.e. differences in mean levels of IL-1β, IL-6, TNF-α, OPG, OPN and OC between PBS and Tris-HCl) and differences in the mean levels of same biomarkers (pg/mL) with the same buffers with or without PI. These comparisons were analyzed using the Mann-Whitney-Wilcoxon test (non-parametric test applied to two independent samples) and Dunn’s Multiple Comparison test as post-hoc analysis.

## Results

Curve adjustment.  Table 2 shows the R2 cytokines curve fitting for the different buffers analyzed (PBS and Tris-HCl with and without PI). All standard curves of biomarkers attained higher values of R2 when using PBS compared to Tris-HCl. When using Tris-HCl, curve adjustment was often low, except for IL-6 and TNF-α, (R2= 0. 9178 and 0.9351 respectively), adjustment curve of the other cytokines did not reach up to R2=0.9 value. All data showed that the use of PBS with PI rendered the best adjustment for IL-6; OPG; OC and TNF-α.

**Table 2:**
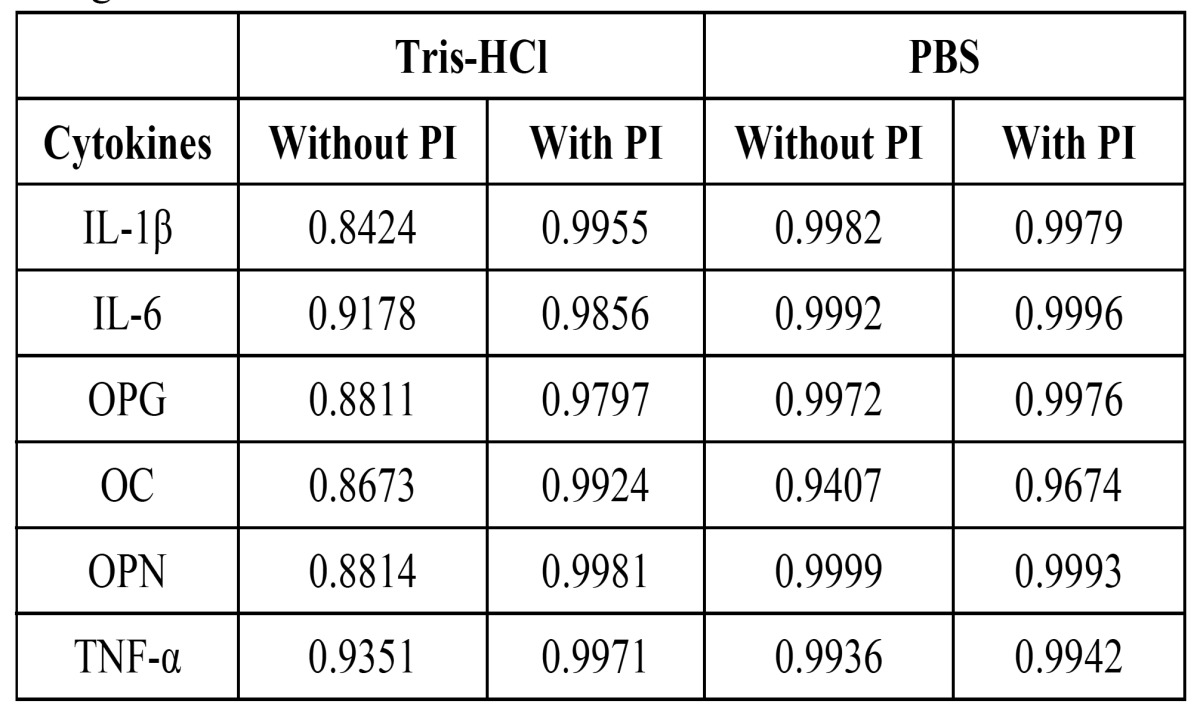
Results obtained of R2 in calibration curves of cytokines using Tris-HCl and PBS with and without PI.

Cytokines Analysis. Cytokines IL-1ß, IL-6 and TNF-α were more often detected when perio -paper strips were diluted with PBS and PI, compared to those diluted with Tris-HCl buffer (*P*= 0.0099; *P*= 0.0371 and *P*= 0.0284, respectively).

In the group treated with PBS buffer, significantly higher levels of IL-6 (*P*< 0.05) were observed when PI was added. For the rest of the cytokines (IL1-ß, OC, OPG and TNF-α), there were also higher levels when adding PI, but differences were not statistically significant (*p*>0.05).

## Discussion

This study shows how positive it is to combine PBS buffer with PI and flow cytometry (Luminex®) techniques to obtain a standard curve and detection/quantification of GCF cytokines. We have tried to achieve high accuracy, and our range (biomarkers into GCF) spans low orders of magnitude, so one way to set up the standard curve was comparing two different buffers adding or not PI into the buffer. During the optimization process, several experimental parameters of importance for the method were detected. The affinity and specificity of the antibody–receptor interactions can be significantly altered because proteins present unwanted interactions depending on buffer chemical properties. Simultaneously, low signal strength provides poor quantitative information, which can be compared when using (or not) protease inhibitors into the buffer. To improve this, PI were added to control unwanted catalytic responses and thus improving the results of the assay. When using Tris-HCL buffer, very low or non-detectable positive signals from IL-1β, OPG, OC and OPN were observed. Analysis of IL-6 and TNF-α rendered variable results with high background and false signals. This result may be due to buffer solubility. On the whole, buffer solubility should be compatible with the soluble protein of interest, coupling receptor chains in unwanted protein-protein interactions and protein-non protein interactions could lead to false signals. As it has been proved, obtaining an optimum curve adjustment by using PBS with PI added (IL-1β, OPG, OPN, IL-6 and TNF-α) also showed high sensitivity leading to better cytokine sample detection. PBS buffer improved the antigen-antibody binding, which was achieved via reactions involving amino groups on the protein and the carboxyl functional groups on the bead surface. Adding PI to PBS rendered the best results detecting specifically IL-1ß, IL-6 and TNF-α versus using PBS without PI. This is probably due to the control of unwanted catalytic responses, improving the amount of proteins and coupling reactions protein, obtaining best coupling conditions showing low background and obtaining reliable simultaneous detection of cytokines. In conclusion, this study has shown that GCF analysis is strongly influenced by the composition of the buffer solution. Using PBS as a buffer with PI added not only increases sensitivity compared to Tris-HCl buffer, but also provides optimal conditions of protein conjugation. PBS buffer and PI can be used to investigate a broad range of cytokines in inflammatory processes.
